# Computational Approach to Musical Consonance and Dissonance

**DOI:** 10.3389/fpsyg.2018.00381

**Published:** 2018-04-04

**Authors:** Lluis L. Trulla, Nicola Di Stefano, Alessandro Giuliani

**Affiliations:** ^1^Centre de Recerca Puig Rodó, Girona, Spain; ^2^Institute of Philosophy of Scientific and Technological Practice and Laboratory of Developmental Neuroscience, Università Campus Bio-Medico di Roma, Rome, Italy; ^3^Environment and Health Department, National Institute of Health, Rome, Italy

**Keywords:** beating, recurrence quantification analysis, complex systems, non-linear signal analysis methods, Devil’s staircase

## Abstract

In sixth century BC, Pythagoras discovered the mathematical foundation of musical consonance and dissonance. When auditory frequencies in small-integer ratios are combined, the result is a harmonious perception. In contrast, most frequency combinations result in audible, off-centered by-products labeled “beating” or “roughness;” these are reported by most listeners to sound dissonant. In this paper, we consider second-order beats, a kind of beating recognized as a product of neural processing, and demonstrate that the data-driven approach of Recurrence Quantification Analysis (RQA) allows for the reconstruction of the order in which interval ratios are ranked in music theory and harmony. We take advantage of computer-generated sounds containing all intervals over the span of an octave. To visualize second-order beats, we use a glissando from the unison to the octave. This procedure produces a profile of recurrence values that correspond to subsequent epochs along the original signal. We find that the higher recurrence peaks exactly match the epochs corresponding to just intonation frequency ratios. This result indicates a link between consonance and the dynamical features of the signal. Our findings integrate a new element into the existing theoretical models of consonance, thus providing a computational account of consonance in terms of dynamical systems theory. Finally, as it considers general features of acoustic signals, the present approach demonstrates a universal aspect of consonance and dissonance perception and provides a simple mathematical tool that could serve as a common framework for further neuro-psychological and music theory research.

## Introduction

Beating is the sensation that typically occurs when two sounds with similar frequencies mutually interfere, giving rise to a waveform with a rhythmic oscillation in amplitude. Following the fundamental contribution of Helmholtz’s treatise, *On the Sensation of Tone* (1954), first published in 1863, contemporary explanations of consonance are grounded in the notions of beating and complex tones—i.e., sounds displaying a broad array of sinusoidal components (harmonics).

Roederer (2008, p. 35) provides an illuminating classification of the effects of superposing two pure tones depending on where in the listener’s auditory system the sounds become entangled. The above mentioned beating is labeled by Roederer as “first-order beating,” because it is processed mechanically in the cochlear fluid and along the basilar membrane. Evidence of the physiological basis of first-order beating stems from the fact that its effect disappears when sounds are played separately in different ears—i.e., dichotically. Another kind of first-order beating effect is known as combination tones, which are produced by the non-linear interaction of waves in narrow spaces, such as the body of musical instruments or the inner ear. Combination tones can be considered as the product of two sine waves. A common example is the *terzo suono* theorized by Giuseppe Tartini (see Lohri, 2016). If *a* and *b* are two frequencies with *a* > *b*, then the *terzo suono* is a tone at frequency *a*–*b* that is discernible only by the listener, because it is produced inside the inner ear rather than being caused by external air vibrations. Combination tones can be heard across the octave at sound pressure levels (SPLs) of 80 dB or higher, and across part of the octave at 50 dB SPL and above.

**FIGURE 1:**
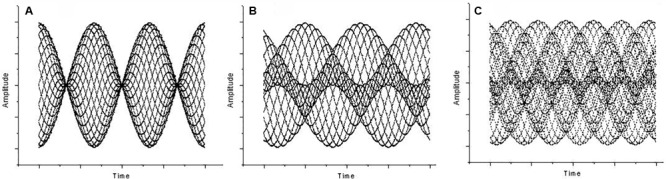
Amplitude (y axis) against time (x axis) for **(A)** Mistuned unison, a case of first-order beating (the interval ratio is 400/403 Hz, 𝜀 = 3, 3 beats). **(B)** Mistuned octave at 803/400 Hz (𝜀 = 3, 3 beats), a case of second-order beating. **(C)** Mistuned fifth at 603/400 Hz (𝜀 = 3, the beats are 2𝜀), also a case of second-order beating.

At 80 dB (or higher) while maintaining the interval around the octave, a distinct beating can be perceived. This disappears when *f*_2_ = 2*f*_1_ (where *f*_1_ and *f*_2_ represent the two frequencies) and reappears as long as the octave becomes mistuned by a factor 𝜀 (i.e., *f*_2_ = 2*f*_1_ + 𝜀). The beating frequency turns out to be 𝜀 (Plomp and Levelt, 1965). Beating “is created by the relatively quick changes produced by modulation frequencies in the region between about 15 to 300 Hz” (Fastl and Zwicker, 2006, p. 257). Unlike first-order beats, the beating persists when tones are fed dichotically, implying that, in this case, beat perception is the result of neural processing. Hence, they are defined as “second-order beats” (Roederer, 2008). Second-order beating shows a modulation in the vibration pattern, i.e., a periodic change in phase difference between the two sounds that form the interval (Roederer, 2008, p. 49), although no amplitude modulation is present. Second-order beats are also called “beats of mistuned consonances” because they are audible when pure tones are superposed to form a fifth (Plomp, 1976). In fact, whereas the vibration pattern of a correctly tuned fifth (*f*_2_ = 3/2 *f*_1_) or fourth (*f*_2_ = 4/3 *f*_1_) is static, the mistuned cases *f*_2_ = 3/2 *f*_1_ + 𝜀 and *f*_2_ = 4/3 *f*_1_ + 𝜀 cause the vibration pattern to change periodically in form, but not in amplitude. From the octave to the fifth and to the fourth, the second-order beats become faster (beating frequency being 𝜀 for the octave, 2𝜀 for the fifth, and 3𝜀 for the fourth) as the vibration pattern grows in complexity (see **Figure 1**).

Their neural origin makes second-order beats an excellent phenomenon for investigating the link between the mathematical description of the signals and their neural processing, and consequently allows us to shed light on their perceived “pleasantness.” To achieve a consistent picture of second-order beats, it is fundamental to overcome the frequency–time space representation trade-off and the related problem of non-stationary signal characteristics.

Graphic representations of sound typically plot the course of amplitude over time or report the relative amplitudes of the different frequencies computed by the Fourier Transform. Thus, there is no mention of time in the latter, and no mention of frequency in the former. However, in the actual hearing process, time and frequency are strictly intermingled, because specific frequencies are processed at specific moments. This fact suggests that we should focus on the simultaneous analysis of time/frequency dimensions (Roads, 2001).

To determine the frequency of an oscillatory phenomenon, we must count the number *n* of vibrations that occur within a set time interval Λ*t*. As *n* is an integer, the minimum error in measuring the frequency is one, thus generating a kind of uncertainty principle in the form Λ*f* ≥ 1/Λ*t*. Increasing the precision of the frequency reclaims a wider window in which to count the time, thus increasing the indetermination of the instant in which the specific frequency occurs.

It is possible to neglect the explicit consideration of time and visualize tone relationships within the octave by computing the ratio of two simultaneous frequencies and then plotting the interval ratio against the amplitude. This is achieved by forming a linear combination of two pure tone waves, a glissando from the unison (*f*_1_) to the octave (2*f*_1_) and a firm wave at frequency *f*_1_. Similar stimuli were previously adopted by Helmholtz (1954) and Kameoka and Kuriyagawa (1969a,b). More recently, Piana (2007) provided a phenomenological explanation of consonance and dissonance when moving from the glissando and ruling out intervals and harmonics. In the following, we propose a numerical approach to Helmholtz’s glissando. Note that this approach maintains the time dimension in terms of the determined sequence of interactions between glissando and the fixed frequency. Focusing on these interactions allows us to overcome the trade-off in the frequency–time representation. For this purpose, we base our approach on the concept of recurrence, a simpler and more fundamental property of the signals with respect to the oscillation frequency (Eckmann et al., 1987; Marwan et al., 2007). The degree of recurrence of a series is estimated by the number of times a signal comes back to an already visited state (see section “Materials and Methods”), and can be computed by the application of recurrence quantification analysis (RQA) (Marwan et al., 2007). Estimating the recurrence rate avoids any stationarity assumption, as the estimate is obtained by a “computation window” sliding along the signal; the result is a profile of recurrence values relative to subsequent epochs along the original signal. This provides a model-free, discrete, and local estimation of the recurrent properties of the series, enabling a quantitative description of second-order beats. The recurrence peaks exactly match the values of the interval ratios corresponding to just intonation and are proportional to the order of consonance of the intervals, thus providing a link between consonance and the dynamical features of the signal.

## Materials and Methods

### Recurrence Quantification Analysis

The original idea of describing non-stationary signals (which are not amenable to classical Fourier analysis) by means of recurrence dates back to the work of Ruelle’s group (Eckmann et al., 1987). The authors introduced recurrence analysis as a purely graphical technique in the form of recurrence plots (RP). Webber and Zbilut (1994) then converted the RP approach into a quantitative technique (RQA) by defining some non-linear descriptors of the RP. RQA has been adopted for the assessment of time series structures in fields ranging from molecular dynamics to physiology and text analysis (Manetti et al., 1999; Orsucci et al., 2006; Marwan et al., 2007). In the field of music research, RQA has been successfully applied to song recognition (Serra et al., 2009) and in the definition of an objective basis of consonance of pure tones (Trulla et al., 2005). In general, this non-linear technique is especially useful for quantifying transient behavior far from the equilibrium (Trulla et al., 1996).

RQA builds upon the computation of a distance matrix between the rows (epochs) of the embedding matrix of the signal of interest, with the lag defined by the method of the first minimum of Mutual Information (Kennel et al., 1992). Given a scalar time series {x(i) = 1; 2; 3;…}, an embedding procedure generates a vector Xi = (x(i); x(i+*L*);…; x(i+(*m*-1)*L*)), where *m* is the embedding dimension and *L* is the lag. {Xi = 1; 2; 3;…; N} then represents the multi-dimensional process of the time series (signal) as a trajectory in *m*-dimensional space. RPs are symmetrical N × N matrices in which a point is placed at (i; j) whenever a point *X*_i_ on the trajectory is close to another point *X*_j_. The relative closeness between X_i_ and X_j_ is estimated by the Euclidian distance between these two vectors. If the distance falls below a threshold radius (*r*), the two vectors (epochs, windows) are considered to be recurrent, and this is graphically indicated by a dot. The value of *r* is usually set to 5–10% of the average pairwise distances between epochs. Therefore, RPs correspond to the symmetrical distance matrix between the epochs (rows of the embedding matrix) of the signal transformed into a binary 0/1 matrix by the action of a threshold.

As an example, consider a time series *A* made up of 10 consecutive values: 7, 8, 10, 15, 6, 7, 9, 11, 10, 8. To observe the recurrence structure of the series at the level of subsequent epochs of length 3, we transform *A* into the embedding matrix *A*E:

**Table:** 

t0	t+1	t+2	epochs
7	8	10	ep1
8	10	15	ep2
10	15	6	ep3
15	6	7	ep4
6	7	9	ep5
7	9	11	ep6
9	11	10	ep7
11	10	8	ep8
			

Thus, the original series has been projected into a three-dimensional space in which the variables (columns) are the time-lagged original series and the statistical units (rows) are the overlapping epochs. The second step is to compute the Euclidean distances between the epochs. This generates the following distance matrix AD:

**Table:** 

ep1	ep2	ep3	ep4	ep5	ep6	ep7	ep8	**TIME**
0								ep1
5.477226	0							ep2
8.602325	10.48809	0						ep3
8.774964	11.35782	10.34408	0					ep4
**1.732051**	7	9.433981	9.273618	0				ep5
**1.41421**	4.242641	8.3666	9.433981	3	0			ep6
3.605551	5.196152	5.744563	8.3666	5.09902	3	0		ep7
4.898979	7.615773	5.477226	5.744563	5.91608	5.09902	3	0	ep8

As the AD elements correspond to the Euclidean distances between corresponding epochs, the diagonal values are 0, and the symmetric character of the distances implies the matrix can be written in lower-triangular form.

We now specify that two epochs are recurrent if their distance is less than 95% of all the between-epoch distances. The average value of the below-diagonal elements of AD is 6.48, and their standard deviation is 2.74. Thus, it is estimated that 95% of distances are greater than 1.74. This implies we have only two recurrences, corresponding to the epoch1–epoch5 and epoch1–epoch6 couples (bolded in the table).

Therefore, example series A has a recurrence rate of 0.071 (two recurrences out of 28 distinct distances) or, equivalently, a recurrence percentage equal to 7.1. The AD matrix corresponds to an RP with only two dots, at coordinates (1, 5) and (1, 6). Note that the recurrences can be identified without the need for any frequency estimation, thus resembling the hearing process that receives sounds as they occur in time.

**FIGURE 2:**
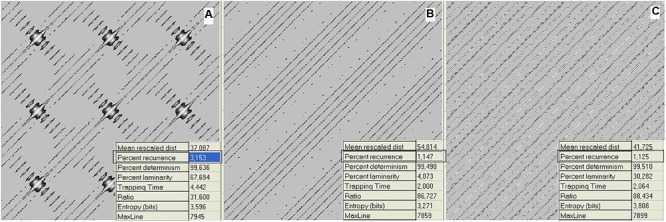
Recurrence plots (RPs) of waveforms for mistuned unison **(A)**, mistuned octave **(B)**, and mistuned fifth **(C)**. Calculations were performed on the data in **Figure 1**. Recurrence algorithm generates several descriptors (inset of the figures) of the recurrence distribution. Here, we consider the recurrence parameter. The axis refers to the discrete timing of the signal. RPs are graphical representations of a between-epochs distance matrix (see section “Materials and Methods”). The main diagonal line refers to the coincidence in time, while increasing distances (along both directions) correspond to the recurrences found at increasing delays. The individual dots denote the epoch pairs that have a distance value below the threshold and are thus considered to be recurrent.

To provide a quantitative measure of the recurrence, numerical RP descriptors were developed (Marwan et al., 2007). We now consider the proportion of recurrent points (dots) in a plot, called the recurrence. Going back to the music domain, **Figure 2** reports the data relative to **Figure 1** as RPs.

### Software

**FIGURE 3:**
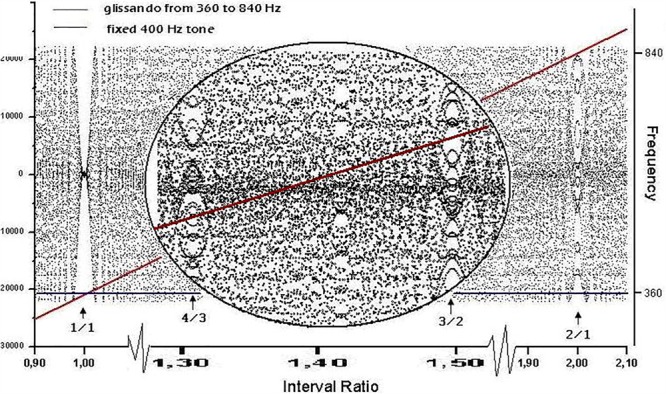
Waveform resulting from linearly adding the amplitudes of two sinusoidal signals: a glissando from 360 to 840 Hz (represented by the diagonal line) and a constant frequency of 400 Hz (line parallel to the x axis). The left y axis shows the amplitude of the waveform and the right y axis is the frequency of the diagonal and plain lines. The x axis shows the time for the glissando to go from 360 to 840 Hz, and therefore contains the full collection of intervals between 360/400 and 840/400. The waveform exhibits a rich texture, as the zoomed inset shows, where the intervals of fourth (4/3) and fifth (3/2) are marked. The discrete character of the signal is the cause of the dot-like nature of the graph. The y axis has both negative and positive numbers depending upon the peak/valley alternation of the combination (where anti-phase destructive interactions correspond to 0).

Files were generated using the sound editor Cool Edit Pro and saved in ASCII format before being fed to the Visual Recurrence Analysis (VRA) software. For the plots in **Figure 1**, we loaded a stereo file of 8000 samples/s to the audio editor, and sent a fixed pure tone of 400 Hz lasting 6 s through the left channel and a fixed pure tone of 403 Hz (**Figure 1A**), 803 Hz (**Figure 1B**), or 603 Hz (**Figure 1C**) for 6 s through the right channel. The sample type was then converted from stereo to mono. **Figure 3** was generated by loading a stereo file of 8000 samples/s to the audio editor, and sending a linearly increasing sound from 360 to 840 Hz lasting 6 s to the left channel and a fixed pure tone of 400 Hz lasting 6 s to the right channel. Finally, the sample type was again converted from stereo to mono.

**Figure 2** shows RPs for the data in **Figure 1**, i.e., 1 s (8000 points) of a mistuned unison, octave, and fifth. The plots were generated by calculating the global recurrence using RQA, as there is no change along the sample. The recurrence of the data shown in **Figure 3** was calculated using a windowing version of an RP, whereby the recurrence is calculated repeatedly for a window that is continuously shifted along the whole sample. Among the RQA parameters, we chose the simplest one, Percent Recurrence, a descriptor that sets the percentage of recurrent points with respect to the non-trivial maximum [equal to (N × (N-1))/2 for an N-point series]. The window for recurrence analysis was 480 points long and the shift was 48 points. The embedding dimension was 5 and the delay was 3 points.

MATLAB programs were obtained from http://sethares.engr.wisc.edu/consemi.html for Sethares’ dissonance curve and http://courses.theophys.kth.se/5A1352/mfiles/devils.m for the theoretical Devil’s staircase (see Discussion).

## Results

A non-stationary signal exploring all interval combinations within the octave can be generated by merging the course of two sounds into a single waveform. The first sound is set at constant frequency *f*_1_ for the full duration of the course, while the second follows an ascending glissando from *f*_1_ to *f*_2_ = 2*f*_1_. **Figure 3** shows an instance of the above procedure.

The most conspicuous singularity (recurrence peaks, see below) in the graph occurs when lines cross themselves, i.e., when *f*_2_ = *f*_1_ (unison, interval ratio of 1:1). A second relevant case occurs at the interval ratio of 2:1, which corresponds to the octave. Less evident events occur at 3:2 (fifth) and 4:3 (fourth), as can be seen in the zoomed inset in **Figure 3**. Singularities in the waveform are thus localized where the frequency ratios are expressed by lower integers and with an apparent amplitude (or degree of singularity) matching the accepted ranking of consonance. In our representation, second-order beats appear as a zone of relative calm centered in rational numbers, surrounded by the tempestuous region of irrationals that Roederer (2008) called “beat holes.”

**FIGURE 4:**
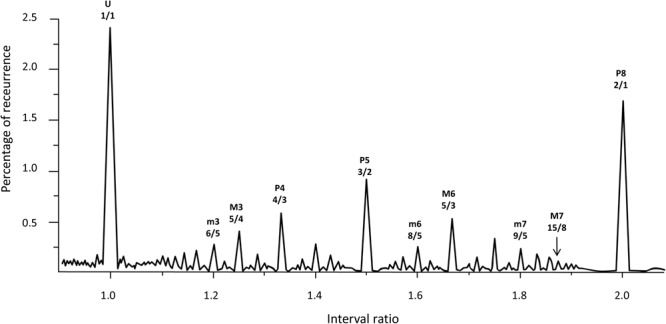
Recurrence analysis of the waveform resulting from linearly adding the amplitudes of sinusoidal signals covering the intervals forming the octave. The x axis is the interval ratio and the y axis gives the percentage of recurrence. Each point in this graph is the result of a single recurrence analysis (like those shown in **Figure 2**), from which we obtain the percentage recurrence. In this case, the RQE algorithm performs recurrence analysis over a window of 480 points, retains the percentage recurrence, slides the 480 point window some 48 points, performs recurrence analysis again, and so on until it exhausts the file. The peaks in the graph are labeled with names and rational numbers according to their position along the x axis interval ratio continuum (see **Table 1** for complete interval list and abbreviations).

Following the numerical solution of Helmholtz’s glissando, we explore the glissando/constant frequency signal through an RQA windowing procedure called Recurrence Quantification of Epochs (RQE). RQE performs a scansion of the whole signal by sequentially selecting small windows—specifically episodes of 480 points—in which the RQA algorithm (with the consequent computation of recurrence rate for each episode) is applied. The subsequent windows are shifted by 48 points and the process is repeated throughout the entire file. For each iteration, we retain both the recurrence value and the interval ratio in which this value occurs, calculated as the mean of the interval ratios in the window. **Figure 4** represents the degree of recurrence along the continuum of interval ratios within the octave.

Emergent features of the glissando are evident in **Figure 4**. Firstly, the higher peaks exactly correspond to the places of just intonation (see Trulla et al., 2005), thus establishing a link between pleasantness and the dynamical features (i.e., recurrence) of the signal. As expected from the numerical model, all peaks correspond to rational numbers. Secondly, it is worth noting the symmetry of the peaks around the perfect fifth. Moreover, the correlation between the extent of recurrence and the rank order of consonance derived from the literature evidences the link between the present model based on signal analysis and results from psychological approaches (see Schwartz et al., 2003, i.e., U > P8 > P5 > P4 > M6 > M3 > m3 > m6 > m7 > M7, in decreasing order of consonance; see **Table 1**).

**Table 1:** Rank order of consonances and their degree of recurrence.

Recurrence	Interval ratio	Label	Rational	Name
100,0	**0.9999**	**U**	**1/1**	**Unison**
89,1	**2.0006**	**P8**	**2/1**	**Octave**
45,2	**1.5003**	**P5**	**3/2**	**Perfect fifth**
30,6	**1.3335**	**P4**	**4/3**	**Perfect fourth**
29,6	**1.6671**	**M6**	**5/3**	**Major sixth**
23,4	**1.2495**	**M3**	**5/4**	**Major third**
19,9	1.7499	H7	7/4	Harmonic seventh
18,5	**1.2003**	**m3**	**6/5**	**Minor third**
16,3	1.4007		7/5	Septimal
15,4	**1.5999**	**m6**	**8/5**	**Minor sixth**
15,1	**1.8003**	**m7**	**9/5**	**Just minor seventh**
14,2	1.1667		7/6	Septimal minor third
11,9	1.2855		9/7	Septimal major third
11,7	1.8339		11/6	Undecimal neutral seventh
11,5	1.1427		8/7	Septimal whole tone
10,1	1.4283		10/7	Euler’s tritone
9,8	**1.1247**	**Mt**	**9/8**	**Major whole tone**
9,7	1.7139		12/7	Septimal major sixth
9,4	1.5711		11/7	Undecimal augmented fifth
9,3	**1.1115**	**mt**	**10/9**	**Minor whole tone**
9,1	1.8567		15/8	Classic major seventh
9,1	1.2219		11/9	Undecimal neutral third
8,7	1.1006		11/10	4/5 tone
8,6	1.3755		11/8	Undecimal semi-augmented fourth
7,8	**1.8747**	**M7**	**15/8**	**Classic major seventh**
7,7	1.2999		13/10	Tridecimal semi-diminished fourth
7,6	1.6251		13/8	Tridecimal neutral sixth
7,1	1.0911		12/11	3/4 tone
6,8	1.8891		17/9	Septendecimal minor third
6,8	1.1823		13/11	Tridecimal minor third
6,7	**1.4451**	**D5**	**13/9**	**Tridecimal diminished fifth**

*Bolded values are intervals most used in Western harmony.*

**FIGURE 5:**
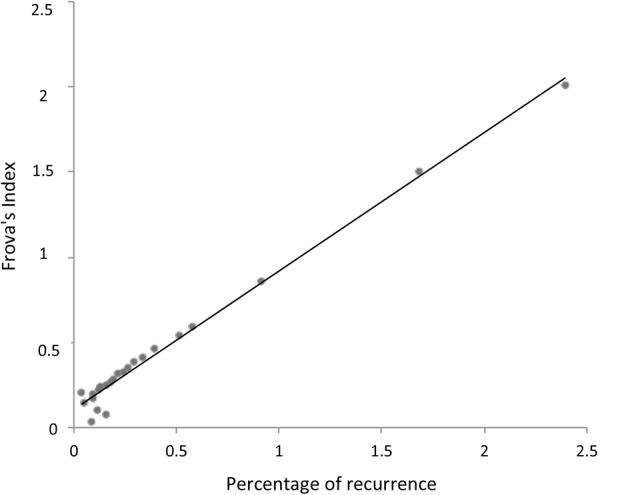
Linear relationship between the degree of recurrence (**Figure 4**) and Frova’s index of consonance (Frova, 1999). Note the almost perfect overlap between the *a posteriori* statistics of actual signals (i.e., recurrence) and the theoretically motivated *a priori* consonance index (i.e., Frova’s index).

In summary, RQA allows us to establish a natural link between the signal properties and the consonance judgment of the listeners without any *a priori* hypothesis or frequency estimation. The reasons why integer numbers play such an important role in harmony has recently been addressed in the literature, with many different recipes presented for calculating the simplicity of the intervals. We use the consonance index provided by Frova (1999) to demonstrate the close relationship between the proposed recurrence index and the bare numerical characteristics of the intervals. If *m*/*n* is the rational number in its lowest terms, Frova’s index is (*m*+*n*)/(*m* × *n*) (Frova, 1999, p. 178). **Figure 5** illustrates the correlation of this index with the notion of simplicity (i.e., degree of recurrence).

Whereas Frova’s index is derived from the energy of the partials forming a complex sound, the percentage recurrence is a purely bottom–up phenomenological descriptor of a pure tone signal, relating recurrence (and consonance) to secondary beating and thus providing a natural (albeit roughly phenomenological) link between the signal properties and neural processing.

**FIGURE 6:**
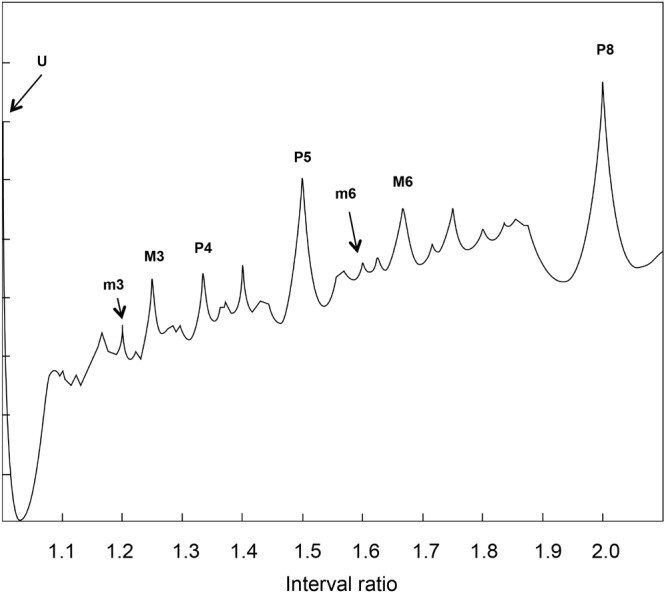
Dissonance curve derived from a synthetic sound with 15 harmonics following a natural series. This graph comes from an algorithm ideated by Plomp and Levelt (1965). The plot is shown upside down for ease of comparison with **Figure 4**. The resemblance between the peaks of **Figure 4** and this figure allows for a straightforward interpretation of recurrence results in terms of consonance/dissonance.

Note that the computation of recurrences gives very similar results with respect to models based on primary beating, such as the Plomp and Levelt model reported in **Figure 6**.

## Discussion

**FIGURE 7:**
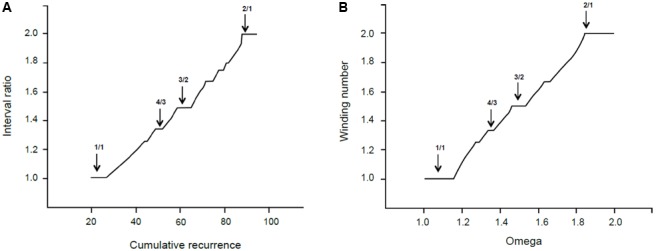
**(A)** Interval ratio vs. cumulative recurrence. **(B)** Theoretical Devil’s staircase from sine map with *k* = 1.

In this paragraph, we relate the self-similar appearance of the recurrence graph in **Figure 4** to the mathematical fractal structures generated by physical processes. **Figure 7** shows the empirical cumulative recurrence distribution (obtained by adding consecutive points) and a formal *Devil’s staircase* in the [1, 2] interval: the similarities between the two graphs are remarkable.

The *Devil’s staircase* pattern is a fingerprint of dynamical systems characterized by the mode-locking phenomenon (Schroeder, 1990, p. 171), which is crucially important in both music generation and perception. In the 17^th^ century, Christian Huygens studied mode-locking and discovered the phenomenon of resonance. He noticed that, after a time, the pendulums of two clocks fixed on the same mounting swung synchronously. The synchronization of two coupled oscillators starting from (slightly) different frequencies is called resonance. A more general case of resonant behavior appears when a specific constant frequency is periodically driven by an external power to oscillate at a different frequency; the so-called Devil’s staircase pattern refers to the behavior of forced *quasilinear oscillators*. In the glissando, the constant frequency is the intrinsic frequency and the glissando the external periodic force. Every plateau in the Devil’s staircase relates to a particular phase-locked solution (stable state), and its relative width forms a hierarchy that follows the explained propriety of rational numbers. The mathematical model for this case is the circle sine map (McCauley, 1994).

(1) $$\theta n+1=\theta n+\frac{p}{q}+(\frac{k}{2\pi })\sin(2\pi \theta n)$$

where *k* is a coupling strength parameter that controls the degree of non-linearity. Without coupling (*k* = 0), the behavior of the system is expressed by the ratio *p*/*q* (often called Ω, the bare winding number). When *k* > 0, the system locks into rational frequency ratios, preferably with small denominators. In this case, the long-term description of the system corresponds to *w*, the dressed winding number. For the critical value *k* = 1, the infinite number of locked frequency intervals corresponding to all the rational numbers between 0 and 1 cover the entire Ω range.

In our terms, Ω is the cumulative recurrence and *w* is the interval ratio. In other words, the system is locked at any rational number—indicated as the interval ratio—but the width or extent of the lock comes from the cumulative recurrence. Thereby, most relevant consonances have extended areas around the lowest rationales—like the unison or octave—and a strong attraction exists toward these exact ratios. This is perfectly sound in terms of music theory.

The above considerations can be summarized in three main points:

(1) A purely empirical, data-driven analysis (RQA) has highlighted a fundamental property of signals (recurrence distribution) that matches the mathematical (number theory) and physical (mode-locking) theoretical background.
(2) The empirical results are consistent with both a theory-driven “simplicity index” (Frova’s index) and with the order that music intervals are ranked in harmony.
(3) The focus on signal properties (second-order beatings) allows us to consider our results as a basis for modeling consonance and dissonance perception by combining data from both computational and cognitive models, e.g., based on artificial neural networks and Hebbian neuroplasticity (Pankovski and Pankovska, 2017).

Numerous studies have confirmed the adequacy of concepts from non-linear dynamics for music perception and construction (e.g., Cartwright et al., 2001, 2002, 2010), and for the study of synchronization among sound sources (Abel et al., 2009). Additionally, neuroscientific research has adopted non-linear dynamical models to describe phase-locked neural populations (Bidelman and Krishnan, 2009; Large and Almonte, 2012) and build *in silico* neuronal models (Lots and Stone, 2008).

Taken together, our work and previous results support the idea that the production and perception of sound are intimately linked, the perceived pleasantness of intervals being an intrinsic property of the signal (in terms of the degree of recurrence), and not only a secondary effect of the signal on the listener. In turn, this allows us to speculate on the auditory system. Second-order beats have been attributed to the central auditory nervous system, and neuronal webs are known to support phase-locking, as in the mammalian auditory system, in which neural activity in areas including the cochlear nucleus, inferior colliculus, and primary auditory cortex is phase-locked to the stimulus waveform (Large and Tretakis, 2005). The mode-locking model was proposed by Lots and Stone (2008) as the basis for musical consonance, leading to the development of a dynamical system model based on stylized neural oscillators producing both synchronization and mode-locking. These results support the idea that both parts of the communication system (the sender and the receiver of sounds) are similarly “wired.” Bidelman and Heinz (2011) applied a waveform to a computational model of the acoustic nerve and, after deriving the autocorrelation function for the nerve fibers, generated the pitch salience profile for the different intervals, giving rise to a distribution that could be superimposed onto the recurrence rate (**Figure 4**). Using an artificial neural network model, Pankovski and Pankovska (2017) recently demonstrated that a specific auditory spectral distribution caused by non-linearities and Hebbian neuroplasticity are sufficient phenomena for a system to generate the consonance pattern.

In line with the literature on music perception (Benade, 1973; Roederer, 2008), we believe that the link between music generation and perception could rely on the fact that the vibrating elements of musical instruments undergo mode-locking into stationary complex vibration patterns. In turn, these can be recognized as the “best fit” to a harmonic template (resident in a properly wired neural circuit). Though this explanation stems from empirical correlations, we are convinced that the simplicity and versatility of the RQA approach could pave the way for neuro-psychological studies with the great advantage of considering the acoustic signal and the perceiver from the same mathematical perspective.

The origins of the distinction between consonance and dissonance have been hotly debated in recent years. As the phenomenon of consonance represents a key element of Western music theory, this has mainly been investigated in terms of Western science (i.e., mathematics, physics, psychoacoustics, and neuroscience). For this reason, Parncutt and Hair (2011) called for studies on the use of consonance and dissonance in non-Western cultures to be conducted in terms of local indigenous musicians, rather than in terms of Western science. In this direction, a relevant study published in *Nature* by McDermott et al. (2016) compares the harmonic preferences of people who have different degrees of exposure to Western music. An indigenous population from Bolivia (the Tsimané) was assumed to have no exposure to Western music, and their preferences were compared with groups of city residents in Bolivia and the United States with different degrees of exposure to Western music. The results show that the subjective preferences of Tsimané participants differ from those of the comparison groups; in particular, they failed to rate consonance as being more pleasant than dissonance. The authors state that, as the Tsimané are able to hear the acoustic distinctions associated with consonance and dissonance, the lack of a measurable preference for consonance appears to reflect difference in their aesthetic response to this contrast (McDermott et al., 2016, p. 549). Correctly, they state that the observed cross-cultural variation suggests that consonance preferences are unlikely to be innate, and so preference is probably acquired. However, the fact that the preference for consonance co-varies with presumptive exposure to Western culture is not sufficient to conclude that consonance perception is not biologically determined. Though preferences vary with cultures, the discrimination of consonance is a prerequisite for preference and has a biological basis, as supported by a large number of neurobiological studies (Koelsch and Mulder, 2002; Koelsch et al., 2005; Minati et al., 2008; Perani et al., 2010; Park et al., 2011; Wang, 2013). Investigating whether consonance perception is biologically determined or shaped by culture is likely to be misleading, as it conceives enculturation as a non-biologically constrained process. Harmonic intervals are a consequence of the entrainment of the nervous system with the sound excitation. This forms a universal biological foundation under any musical culture, determining the distinction between acoustic consonance and dissonance and leaving it to each culture to determine exactly how to employ these acoustic distinctions. However, the existence of different musical cultures and systems does not imply the lack of a shared natural/biological basis for music production. The interaction between nature and culture is much more complex, and cross-cultural variations in musical systems only show that biology does not rigidly determine music aesthetics. Similar considerations have led to a more adequate definition of music as a “biocultural phenomenon” (Cross, 2003).

## Conclusion

The main contribution of this paper stems from the numerical solution of Helmholtz’s glissando. Though the standard modern theory of consonance is based on first-order beating, we have shown that similar results can be obtained starting from second-order beats. The recent interest in second-order beating has been fruitful for models of pitch recognition or neural circuitry (see Roederer, 2008), but not for theories on consonance.

Scholars have started to consider music from the perspective of dynamical systems, both in neurobiological and physical terms, showing that mode-locking models can explain how the nervous system manages sound and is engaged in the ranking of consonances. The resemblance between the formal Devil’s staircase model and the cumulative recurrence distribution strengthens this idea.

From a methodological perspective, the main contribution of this work is to provide neuroscience scholars with an extremely simple and model-free tool (RQA) that approaches the acoustic signal and the listener’s perception system with the same mathematical method. Different RQA applications have been reported in research on otoacoustic emission (see, for example, Zimatore et al., 2002, 2003). We are therefore confident that the use of a simple statistical approach will foster interactions between music theory and neuro-psychological approaches.

Finally, our results support the idea of natural roots of consonance perception, and are thus in line with several studies published in recent years (see, for example, Wang, 2013; Bowling and Purves, 2015; Nikolsky, 2015; Foo et al., 2016; González-García et al., 2016; Di Stefano et al., 2017). However, as proved by McDermott et al. (2016), the role of perception in the formulation of aesthetic judgment remains unclear. Therefore, musical consonance and dissonance remains a hotly debated topic (see Bowling et al., 2017), in need of further research to merge different approaches into a consistent theory.

## Author Contributions

LT originally conceived the idea of the paper, elaborated the stimuli, provided all the figures, and significantly contributed to the results and discussion. NDS prepared the manuscript, co-authored the introduction and the results with LT, contributed to the discussion, wrote the conclusion, and finally revised the entire draft. AG wrote the section “Recurrence Quantification Analysis,” reviewed the entire manuscript, and suggested useful ideas for the discussion. All authors equally contributed to the revision of the manuscript before agreeing on the final version.

## Conflict of Interest Statement

The authors declare that the research was conducted in the absence of any commercial or financial relationships that could be construed as a potential conflict of interest.
